# Robotic re-TAPP: a minimally invasive alternative for the failed posterior repair

**DOI:** 10.1590/0100-6991e-20223063

**Published:** 2022-02-18

**Authors:** PEDRO HENRIQUE DE FREITAS AMARAL, LUCA GIOVANNI ANTONIO PIVETTA, EDUARDO RULLO MARANHÃO DIAS, JOÃO PAULO VENANCIO DE CARVALHO, MARCELO FURTADO, CARLOS ALBERTO MALHEIROS, SERGIO ROLL

**Affiliations:** 1 - Hospital Alemão Oswaldo Cruz, Centro de Hérnia - São Paulo - SP - Brasil; 2 - Santa Casa de São Paulo, Grupo de Parede Abdominal e Cirurgia Bariátrica - Departamento de Cirurgia - São Paulo - SP - Brasil

**Keywords:** Hernia, Inguinal, Robotic Surgical Procedures, Recurrence, Minimally Invasive Surgical Procedures, Hérnia Inguinal, Robótica, Recidiva, Procedimentos Cirúrgicos Minimamente Invasivos

## Abstract

**Objective:**

to describe the use of the robotic platform in inguinal hernia recurrence after a previous laparoscopic repair.

**Methods:**

patients with recurrent inguinal hernias following a laparoscopic repair who have undergone robotic transabdominal preperitoneal between December 2015 through September 2020 were identified in a prospectively maintained database. Outcomes of interest included demographics, hernia characteristics, operative details and rates of 30-day surgical site occurrence, surgical site occurrences requiring procedural interventions, surgical site infection and hernia recurrence were abstracted.

**Results:**

nineteen patients (95% male, mean age 55 years, mean body mass index 28) had 27 hernias repaired (N=8 bilateral). Average operative time was 168.9 ± 49.3min (range 90-240). There were two intraoperative complications all of them were bleeding from the inferior epigastric vessel injuries. Three SSOs occurred (N=2 seromas and N=1 hematoma. After a median 35.7 months follow-up (IQR 13-49), no recurrence has been diagnosed. One patient developed chronic postoperative inguinal pain.

**Conclusions:**

on a small number of selected patients and experienced hands, we found that the use of the robotic platform for repair of recurrent hernias after prior laparoscopic repair appears to be feasible, safe and effective despite being technically demanding. Further studies in larger cohorts are necessary to determine if this technique provides any benefits in recurrent inguinal hernia scenario.

## RESUMO

**Objetivo:**

descrevemos nossa experiência com uso da plataforma robótica no tratamento das recidivas operadas previamente por laparoscopia, mantendo assim uma proposta minimamente invasiva a esses pacientes, apesar de haver uma predileção pela via anterior e aberta nestes casos.

**Métodos:**

foram incluídos pacientes submetidos a hernioplastia inguinal robótica transabdominal pré-peritoneal como tratamento de recidiva e que foram operados previamente por laparoscopia, entre dezembro de 2015 e setembro de 2020 e mantidos em uma base de dados ambulatorial prospectiva. Variáveis de interesse incluíram dados demográficos, características herniárias, detalhes operatórios, ocorrências do sítio cirúrgico em 30 dias (com ou sem necessidade de intervenção), infeção do sítio cirúrgico, tempo de seguimento e taxa de recidiva.

**Resultados:**

dezenove pacientes (95% masculino, média de idade de 55 anos, média de índice de massa corporal 28kg/m^2^) e 27 hérnias operadas (N=8 bilaterais). Média de tempo cirúrgico 168.9±49.3 min (variando 90-240). N=2 complicações intraoperatórias por lesão de vasos epigástricos inferiores. N=2 seromas e N=1 hematoma foram identificados no pós-operatório; N=1 paciente apresentou dor crônica pós operatória. Após um tempo de seguimento médio de 35.7 meses (intervalo entre quartis 13-49), nenhuma recidiva foi diagnosticada.

**Conclusões:**

o uso da plataforma robótica parece ser seguro e efetivo no tratamento das recidivas operadas previamente laparoscopia, nesse pequeno grupo de pacientes selecionados, apesar de requerer expertise em cirurgia robótica. Outros estudos com maiores casuísticas são necessários para estabelecer o papel desta técnica no cenário das hérnias inguinais recidivadas.

## INTRODUCTION

Laparoscopic inguinal hernia repair is associated with shorter recovery time, less postoperative pain and equivalent long-term recurrence rates, when compared to traditional open mesh-based repair^1^ ^,^ ^2^. Recently, Dominguez et al.^3^ described the implementation of the robotic platform to perform a transabdominal preperitoneal (r-TAPP) inguinal hernia repair. Improved ergonomics, three-dimensional visualization and increased dexterity for dissection and suturing the mesh in place were deemed potential benefits of the robot for inguinal hernia repair. Traditionally, recurrence after a prior posterior approach is repaired using an anterior approach, as a prior violation of the preperitoneal space can lead to scar tissue formation making the repair challenging. The Brazilian Hernia Society Guidelines^4^ endorse the recommendation from the European^5^ and International Hernia Societies^2^ when dealing with a recurrence after a TAPP, the patient should be treated with the anterior open repair. However, we hypothesized that the aforementioned benefits of the robotic platform might facilitate a re-do TAPP in selected cases, that is an opportunity to offer minimally invasive treatment to patients who had posterior approach failure. Furthermore, this can also be done to patients who had already had an approach by open anterior and laparoscopic posterior approach.

 We aimed to report our medium-term results with r-TAPP in patients who failed previous laparoscopic repair.

## METHODS

After obtaining institutional ethics committee approval (CAAE: 81843817.6.0000.0070 / approved by Opinion 4.467.803), all patients who undewent a r-TAPP repair due to recurrent inguinal hernias and had previously been operated by laparoscopic were retrospectively identified in a prospectively maintained database. The procedures were performed by the senior author in a single center, in a private setting, in São Paulo/Brazil - Hospital Alemão Oswaldo Cruz. Patients were operated between December 2015 through September 2020.

Patient demographics, hernia characteristics, operative details and 30-day surgical site occurrence (SSO), surgical site occurrences requiring procedural interventions (SSOPI), surgical site infection (SSI) and hernia recurrence rates were retrieved from the database. Hernia characteristics were graded according to the European Hernia Society groin hernia classification^6^.

Wound events were reported using standard definitions. SSIs were classified according to the Centers for Disease Control and Prevention^7^ (CDC) classification as superficial, deep incisional and organ space. SSOs include wound cellulitis, non-healing wound, skin and soft tissue ischemia or necrosis, wound serous drainage, seroma, hematoma, exposed mesh or enterocutaneous fistula. SSOPIs include any SSO that required wound opening or debridement, suture excision, percutaneous drainage and partial or complete mesh removal. 

Descriptive statistics were used to report data, using counts and percentages, means, standard deviations or medians and interquartile ranges as appropriate.

### Surgical Technique

Patient preparation: Surgery was performed under general anesthesia. Antibiotic prophylaxis with a first general cephalosporin was administered during anesthesia induction. Bladder was routinely decompressed by insertion of a Foley catheter after induction of anesthesia. All patients received a combination of mechanic and pharmacological prophylaxis of venous thromboembolic events according to institutional protocols. 

**Figure 1:**
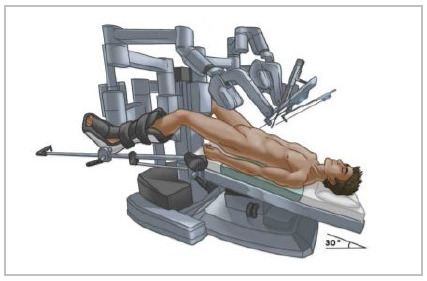
Patient positioning using legs support and robot between the legs.

**Figure 2:**
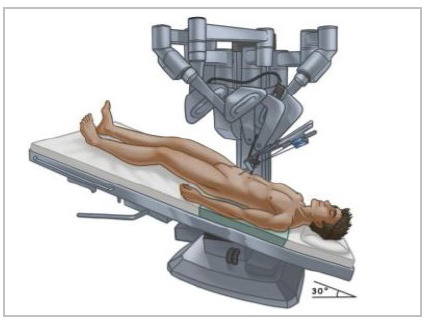
Patient in supine positioning and lateral docking.

Patient positioning: The patient was placed in supine position and both arms tucked. The robot platform was placed between the legs using legs support (Figure 1) or laterally with the legs extended (Figure 2). After docking, the surgical table was positioned on a slight Trendelenburg position. 

**Figure 3:**
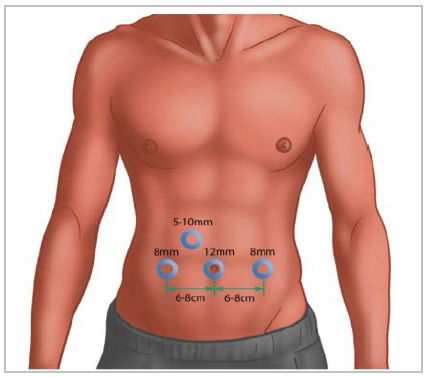
Ports placement scheme.

Surgical procedure: The peritoneal cavity was accessed at the umbilicus using cut-down technique. The abdomen was insufflated, the laparoscope was inserted. Three additional robotic ports were placed being two 8mm ports bilaterally in line with de umbilicus at a distance of 6 to 8cm and an auxiliary 5mm or 10mm port was placed behind (Figure 3). All procedures were performed using DaVinci Si^®^ - Intuitive.

**Figure 4:**
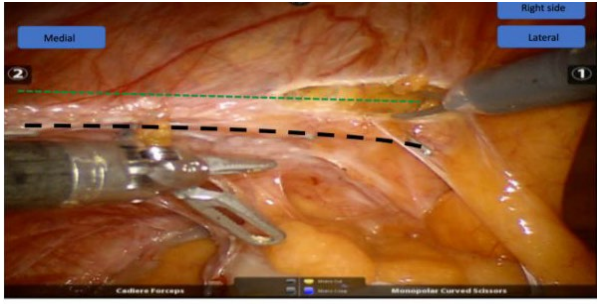
Peritoneal flap creation: the incision should be done right above (green line) the previous incision da linha (black line), avoiding the fibrosis.

**Figure 5:**
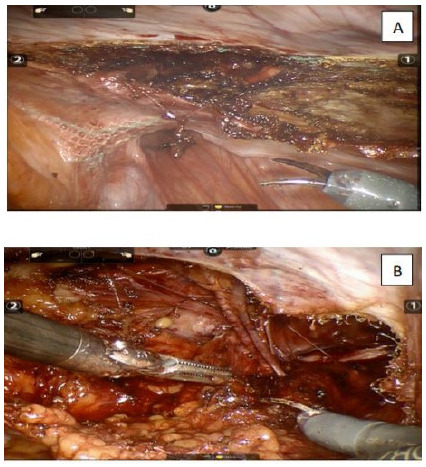
fibrosis between the mesh, peritoneum and epigastric vessels (Figures 5A e 5B).

**Figure 6:**
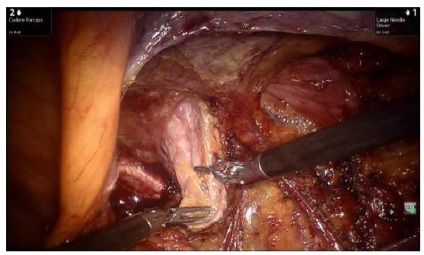
Medial hernia sac dissected.

**Figure 7:**
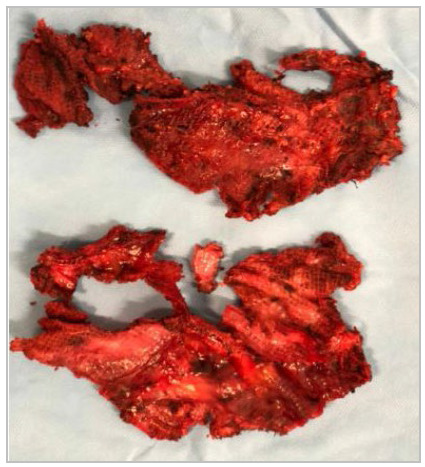
Mesh removal.

Adhesions were taken down and visceral incarceration was reduced when necessary. Dissection of the peritoneal flap was started more cranially avoiding, initially, the area with scar tissue from the prior operation (Figure 4). Dissection can be oftentimes challenging due to fibrosis in the mesh-peritoneum interface (Figures 5A and 5B). Eventual hernia sac is identified, dissected free off the chord structures on a medial to lateral fashion and reduced (Figure 6). The preperitoneal pocket dissection should be wider than what was accomplished on the original procedure to ensure wide mesh overlap. Complete mesh removal was attempted in all cases (Figure 7). In the cases of intense fibrosis posing neurovascular structures at risk for damage, partial mesh removal was performed leaving a rim of mesh around the spermatic chord. Complete dissection of the myopectinal orifice (MPO) was performed as described by Felix et al^8^. 

**Figure 8:**
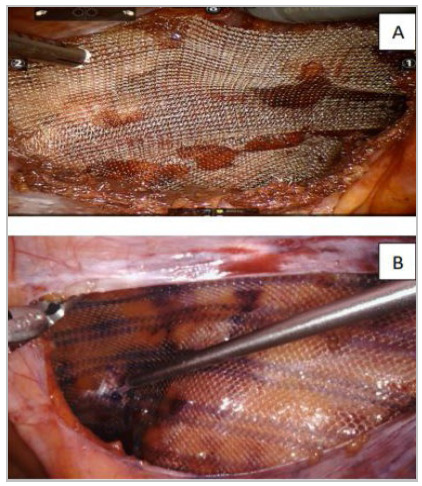
Mesh fixation using tacker (A) or tissue adhesive (B).

Polypropylene mesh was used with a minimum 12x15cm dimensions. Mesh was rolled and inserted in the cavity through the 10mm auxiliary port or 8mm port and placed into the pocket covering the MPO. Mechanical fixation with a tacker (Securestrap - Johnson&Johnson^®^) or atraumatic mesh fixation with tissue adhesives were used. (Histoacryl - B Braun^®^) (Figures 8A and 8B). The peritoneal flap was closed with running barbed suture (15cm, V-Lok - Medtronic^®^/ Stratafix - Johnson&Johnson^®^) . More details of the r-TAPP are reported by Podolsky et al.^9^.

## RESULTS

Nineteen patients were identified. Eight had bilateral recurrent hernias, totalizing 27 hernias repaired. The majority were male (N=18; 94.7%) of the patients with a mean age of 55.2 years (± 13.1). Table 1 presents demographic information, and operative details. The mean body mass index was 28.1±4, (19.4-32.9) kg/m^2^, (SD, range). All patients were CDC wound class 1. Average operative time was 168.9±49.3min (range 90-240). There were two intraoperative complications (10.5%), all of them were bleeding from the inferior epigastric vessel, injuries that were controlled with clips. 

**Table 1:** Demographic information and operative details.

Demographic	n (%)
Age, years, mean±SD (range)	55.2±13.1 (32-72)
Male gender	18 (94,7)
Body mass index, kg/m^2^ mean±SD (range)	28.1±4 (19.4-32.9)
Hypertension	4 (21.1)
Obesity	6 (31.5)
Diabetes	2 (10.5)
Smoking	2 (10.5)
Former smoking	2 (10.5)
CDC wound Class 1 (clean)	19 (100)
Operative details	
Mean operative time, minutes±SD (range)	168.9±49.3 (90-240)
Intraoperative complications	2 (10.5)
Peritoneal closure with continuous barbed suture	19 (100)

The majority were recurrences after TAPP (N=18; 94.7%). Only one was recurrence after the Totally Extraperitoneal (TEP) approach. Three cases were recurrent hernias after both posterior and anterior approaches. Considering hernia classification, recurrent medial were observed in N=16 hernias, recurrent lateral hernia were observed in N=9 hernias, combined medial and lateral were observed in N=1 hernia and N=1 femoral hernia. The mesh was completely removed in 4 cases (21.1%) and partially removed in the remaining cases. With respect to mesh fixation, a tacker was used in N=10, tissue adhesive in N=8 and both techniques in N=1. Heavyweight mesh was chosen in N=13 cases, midweight in N=5 cases and lightweight in N=1 case. None of the 27 repairs were converted to open operation. Hernia and mesh information are described in Table 2. 

**Table 2:** Hernia and mesh information.

patient	hernia type	EHS classification	previous repair	mesh choice
# 1	left	Mr1	TAPP	heavyweight
# 2	left	Lr2	TAPP	heavyweight
# 3	right	Mr2	TAPP	heavyweight
# 4	bilateral	Lr3 (left) / Lr1 (right)	TAPP	heavyweight
# 5	bilateral	Lr2 (left) / Lr2 (right)	TAPP	heavyweight
# 6	right	Mr2	TAPP	heavyweight
# 7	right	Lr2	TEP	lightweight
# 8	bilateral	Mr1 (left) / Mr1 (right)	TAPP	heavyweight
# 9	left	Mr1	TAPP	midweight
# 10	bilateral	Mr2 (left) / Mr2 (right)	Lichtenstein / TAPP	heavyweight
# 11	bilateral	Mc (left) / Mr2 (right)	Lichtenstein / TAPP	heavyweight
# 12	right	Lr2	TAPP	heavyweight
# 13	bilateral	Lr2 (left) / Lr2 (right)	Lichtenstein / TAPP	heavyweight
# 14	right	Mr3	TAPP	heavyweight
# 15	right	F	TAPP	heavyweight
# 16	bilateral	Mr2 (left) / Mr2 (right)	TAPP	midweight
# 17	bilateral	Mr2 (left) / Mr3 (right)	TAPP	midweight
# 18	right	Mr2	TAPP	midweight
# 19	left	Mr2	TAPP	midweight

At 30-day follow-up, three SSO’s were identified. Two patients had a seroma and one patient had a hematoma that were managed without any intervention. After a median 35.7 months follow-up (IQR 13-49), no recurrence has been diagnosed. One patient complained of persistent groin pain in the pubic bone with radiation to the testicle. This patient was managed with pain medications and no interventional pain management was required. The outcomes are summarized in Table 3. 

**Table 3:** Surgical outcomes.

Outcome	n (%)
Median hospital stay, days (IQR)	1 (1-2)
30-day SSO	3 (15.7)
seroma	2 (10.5)
hematoma	1 (5.2)
30-day SSOPI	0
30-day SSI	0
unplanned readmission	0
Hernia reccurence	0
Median follow-up	35.7mouths
	IQR 13 - 49

## DISCUSSION

The laparoscopic inguinal hernia repair emerged as the procedure of choice over conventional open techniques due to well-documented advantages such as lower rates of postoperative pain, shorter return time to activities, lower incidence of infections, less chronic inguinal pain and comparable recurrence rates^10,11.^

Even though the Brazilian^4^, European^5^ and International guidelines^2^ suggest alternating the approach for recurrence treatment, other authors have reported encouraging results with reoperation through a posterior approach, maintaining minimally invasive treatment^12^ ^-^ ^14^. More recently, Fernandez-Alberti et al.^15^ published a comparative study in which their laparoscopic recurrences were divided into two possible treatments: Lichtenstein and re-TAPP. In this study, re-TAPP surgery for recurrences after previous TAPP repair indicated shorter hospital stay and lower morbidity while there were comparable recurrence rates.

In our cohort of 19 patients and 27 operated hernias, some intraoperative considerations deserve attention. From these cases, we observed that recurrences occurred in two different ways. (1) Recurrence below the mesh - when the mesh dislodges cranially, which may be medial or lateral to the epigastric vessels or (2) when an inadequate mesh fixation or insufficient mesh overlap results in extrusion of the mesh into the direct or indirect defect. Heuvel and Dwars14 have reported similar findings. 

We recommend placing a new mesh with dimensions of at least 15x12cm. The largest one used in this study had 20x15cm, which guarantees a wide overlap, from the iliac crest to the midline. Fernandez-Alberti et al.^15^, Deans et al.^16^, and Leibl et al.^17^ had already suggested that the placement of wider meshes is related to lower re-recurrences rates. In this sense, we believe that the use of the robotic platform facilitates more extensive and safer dissections, including in the regions of fibrosis and the presence of previous mesh. 

Felix et al.^18^ reoperated 33 patients after a laparoscopic repair and completed the repair with a TAPP technique in all patients; however, in four cases, they used a combined approach (laparoscopic and anterior). Heuvel and Dwars^14^ completed the re-TAPP by the laparoscopic approach in 96.2%. In our series, there was no need for conversion to the anterior open approach, but we suggest it is pertinent to consider the change of route - to anterior - if, after endoscopic exploration, a potentially threatening risk for the patient is identified, as reported by Kockerling et al.^19^.

Concerning the incidence of pain in the postoperative period, only one patient (5,2%) presented this symptom, without the need for anesthesia block or surgery, being adequately treated with medication, and presenting pain remission. This result corroborates with Kockerling et al.^12^ that presented a lower incidence of pain in re-TAPP using laparoscopic approach. Nienhuijs et al.^20^ reported in their review an incidence of chronic pain of 11%, and Langeveld et al.^21^ reported that chronic pain one year after laparoscopic or open inguinal hernia repair is seen in 25% of the cases. The variety of results concerning chronic pain in the series suggest that the chronic pain after hernia repair can be underestimated and underdiagnosed.

Despite its costs, there are some of the advantages of the robotic platform when repairing recurrent hernias after a posterior approach. In our opinion, those are enhanced three-dimensional visualization that allows more precise dissection of the mesh-peritoneum interface, especially around the bladder and iliac vessels. Similarly, improved ergonomics and surgical dexterity contribute to shortening the operative time on complex dissections, as highlighted by the average operative time seen in our series (168.9 minutes), which included docking and unilateral or bilateral repairs.

In three cases, the patients had a first laparoscopic repair, had a recurrence, and underwent open repair - Lichtenstein, directed by the guidelines but had a recurrence. The recurrence rate in the first repair is low, however there are increasing rates of recurrence in subsequent repairs. In these specific re-recurrence cases, in whom both approaches (anterior and posterior) were performed, the guidelines recommendations are not clear to guide the surgeon’s choice (re-Lichtenstein, re-TAPP, or robotics). Furthermore, the recurrence procedures are demanding operations that should be done in high specialized centers for abdominal wall surgery^22^. Lydeking et al.^23^ documented in a multi-center prospective single-blinded, randomized trial on TAPP vs. Lichtenstein’s repair in male patients operated for a recurrent inguinal hernia after a primary open inguinal hernia repair that the long-term re-recurrence rate and chronic pain incidence were surprisingly high respectless of the surgical approach. Neither TAPP nor Lichtenstein’s procedure was superior to improve surgical results. Even though there are no apparent benefits, we prefer to keep the treatment minimally invasive as it offers patients an earlier return to activities. To our knowledge, the present study is the first cohort using robot assisted TAPP re-do after failed posterior laparoscopic approach (N=18 TAPP; N=1 TEP). 

Our study has several limitations that deserve mention. Despite the outcomes after r-TAPP for failed posterior hernia repair were satisfactory in our hands, our data is limited to a single surgeon with extensive experience in robotic surgery and these results might not be repeated in other units. Although data were prospectively collected, our study is retrospective in nature since it involves a review of prospectively maintained database. Additionally, accurate statistic tests are not recommended for small series of cases. Until the present moment, there are few reports in the literature studying the posterior approach as a reoperation, and there are no reports with robotic surgery in this context, what limits the basis to determine the sample size. Accordingly, more studies with a larger number of patients, other surgeons and a well-matched control group are necessary to provide meaningful conclusions.

## CONCLUSION

A small number of selected patients in experienced hands indicated the use of the robotic platform for repair of recurrent hernias after prior laparoscopic repair to be feasible, safe and effective despite being technically demanding. Larger cohort studies, with other surgeons’ experience are necessary to determine if this technique provides any benefits in recurrent inguinal hernia scenario.

## References

[1] Wu JJ, Way JA, Eslick GD, Cox MR (2018). Transabdominal Pre-Peritoneal Versus Open Repair for Primary Unilateral Inguinal Hernia A Meta-analysis. World J Surg.

[2] HerniaSurge Group (2018). International guidelines for groin hernia management. Hernia.

[3] Escobar Dominguez JE, Gonzalez A, Donkor C (2015). Robotic inguinal hernia repair. J Surg Oncol.

[4] Claus CMP, Oliveira FMM, Furtado ML, Azevedo MA, Roll S, Soares G, Nacul MP (2019). Guidelines of the Brazilian Hernia Society (BHS) for the management of inguinocrural hernias in adults Rev. Col. Bras. Cir.

[5] Miserez M, Peeters E, Aufenacker T, Bouillot JL, Campanelli G, Conze J (2014). Update with level 1 studies of the European Hernia Society guidelines on the treatment of inguinal hernia in adult patients. Hernia.

[6] Miserez M, Alexandre J, Campanelli G, Corcione F (2007). The European Hernia Society groin hernia classification simple and easy to remember. Hernia.

[7] Berríos-Torres SI, Umscheid CA, Bratzler DW, Leas B, Stone EC, Kelz RR (2017). Healthcare Infection Control Practices Advisory Committee Centers for Disease Control and Prevention Guideline for the Prevention of Surgical Site Infection, 2017. JAMA Surg.

[8] Daes J, Felix E (2017). Critical View of the Myopectineal Orifice. Ann Surg.

[9] Podolsky D, Novitsky Y (2020). Robotic Inguinal Hernia Repair. Surg Clin North Am.

[10] Liem MS, van Duyn EB, van der Graaf Y, van Vroonhoven TJ, Coala Trial Group (2003). Recurrences after conventional anterior and laparoscopic inguinal hernia repair a randomized comparison. Ann Surg.

[11] Ertem M, Ozben V, Gok H, Ozveri E (2013). Relaparoscopic treatment of recurrences after previous laparoscopic inguinal hernia repair. Minim Invasive Surg.

[12] Köckerling F, Bittner R, Kuthe A, Stechemesser B, Lorenz R, Koch A (2017). Laparo-endoscopic versus open recurrent inguinal hernia repair should we follow the guidelines?. Surg Endosc.

[13] Eklund A, Rudberg C, Leijonmarck CE, Rasmussen I, Spangen L, Wickbom G (2007). Recurrent inguinal hernia randomized multicenter trial comparing laparoscopic and Lichtenstein repair. Surg Endosc.

[14] van den Heuvel B, Dwars BJ (2013). Repeated laparoscopic treatment of recurrent inguinal hernias after previous posterior repair. Surg Endosc.

[15] Fernandez-Alberti J, Iriarte F, Croceri RE, Medina P, Porto EA, Pirchi DE (2021). Laparoscopic treatment (reTAPP) for recurrence after laparoscopic inguinal hernia repair. Hernia.

[16] Deans GT, Wilson MS, Royston CM, Brough WA (1995). Recurrent inguinal hernia after laparoscopic repair possible cause and prevention. Br J Surg.

[17] Leibl BJ, Schmedt CG, Kraft K, Ulrich M, Bittner R (2000). Recurrence after endoscopic transperitoneal hernia repair (TAPP) causes, reparative techniques, and results of the reoperation. J Am Coll Surg.

[18] Felix E, Scott S, Crafton B, Geis P, Duncan T, Sewell R (1998). Causes of recurrence after laparoscopic hernioplasty A multicenter study. Surg Endosc.

[19] Köckerling F, Schug-Pass C (2017). Diagnostic Laparoscopy as Decision Tool for Re-recurrent Inguinal Hernia Treatment Following Open Anterior and Laparo-Endoscopic Posterior Repair. Front Surg.

[20] Nienhuijs S, Staal E, Strobbe L, Rosman C, Groenewoud H, Bleichrodt R (2007). Chronic pain after mesh repair of inguinal hernia a systematic review. Am J Surg.

[21] Langeveld HR, van't Riet M, Weidema WF, Stassen LP, Steyerberg EW, Lange J (2010). Total extraperitoneal inguinal hernia repair compared with Lichtenstein (the LEVEL-Trial) a randomized controlled trial. Ann Surg.

[22] Stabilini C, Cavallaro G, Bocchi P, Campanelli G, Carlucci M, Ceci F (2018). Defining the characteristics of certified hernia centers in Italy The Italian society of hernia and abdominal wall surgery workgroup consensus on systematic reviews of the best available evidences. Int J Surg.

[23] Lydeking L, Johansen N, Oehlenschläger J, Bay-Nielsen M, Bisgaard T (2020). Re-recurrence and pain 12 years after laparoscopic transabdominal preperitoneal (TAPP) or Lichtenstein's repair for a recurrent inguinal hernia a multi-centre single-blinded randomised clinical trial. Hernia.

